# Obesity, a Single Pathology Influencing Both Mother and Child—A Retrospective Analysis in Hospital Settings

**DOI:** 10.3390/jpm14070683

**Published:** 2024-06-26

**Authors:** Cristina Mihaela Ormindean, Razvan Ciortea, Carmen Elena Bucuri, Andrei Mihai Măluțan, Cristian Ioan Iuhas, Ciprian Gheorghe Porumb, Vlad Ormindean, Maria Patricia Roman, Ionel Daniel Nati, Viorela Suciu, Dan Mihu

**Affiliations:** 2nd Department of Obstetrics and Gynaecology, “Iuliu Hatieganu” University of Medicine and Pharmacy, 400012 Cluj-Napoca, Romania; cristina.mihaela.prodan@gmail.com (C.M.O.); cbucurie@yahoo.com (C.E.B.); vladormindean@gmail.com (V.O.);

**Keywords:** obesity, pregnancy, labor, delivery, mechanisms of obesity, fetal distress

## Abstract

Obesity, characterized by an excess of adipose tissue, has become a significant global health issue. The prevalence of obesity has increased markedly in recent decades worldwide, with a sharp rise also observed in developing countries, particularly in urban areas. Addressing obesity during pregnancy is crucial for several reasons and presents challenges for specialists in obstetrics and gynecology. Objectives: The aim of the present study was to investigate the correlation between obesity and its implications for childbirth. Materials and Methods: We conducted a retrospective study involving 1513 patients, grouped into normal-weight, overweight, and obese categories using corrected BMI values. We performed comparative analyses to explore the association between BMI and various outcomes: the method of delivery, the Apgar score at birth, the incidence of fetal distress, fetal birth weight, the presence of pregnancy-associated pathologies, and the occurrence of postpartum hemorrhage. Descriptive statistical analysis was utilized to characterize the demographic and clinical features of the patients and newborns. Results: By examining variables such as the occurrence of fetal distress during labor, the Apgar score at delivery, and the mode of delivery, we identified an association between increasing BMI and complications during labor and delivery. The results indicate that a higher BMI is linked with increased complications and variations in the mode of delivery. Conclusions: Obesity is the most common health issue among women of reproductive age and requires long-term care. It can contribute to numerous pregnancy-associated pathologies and affect both mother and child during labor and delivery. Obesity is associated with lower Apgar scores, the increased incidence of fetal distress, and a higher rate of cesarean section deliveries. Although the absolute risk of serious complications for mother, fetus, and newborn is low among women with obesity, adopting healthy eating and exercise behaviors prior to pregnancy, ideally, or as early in pregnancy as possible, can help minimize excessive weight gain during pregnancy.

## 1. Introduction

Obesity, characterized by an excess of adipose tissue, has become a significant global health problem. It is commonly assessed using body mass index (BMI), with a BMI of 30 or higher categorizing the patient as obese. In 2005, the World Health Organization (WHO) estimated that approximately 700 million adults would become obese by 2015 [1,2]. The prevalence of obesity has increased significantly in recent decades globally, and this trend is also evident among women of reproductive age (15–44 years), reaching 100 million, with a further 250 million women classified as overweight [3,4], making obesity in pregnancy a critical public health issue. While the prevalence is higher in developed countries, rates have also risen sharply in developing countries, particularly in urban areas [4,5].

To fully understand the implications of obesity in pregnancy, it is essential to comprehend this pathology and its evaluation. Obesity is not merely being overweight; it is a complex condition that can affect the body in numerous ways, influencing metabolic health, hormonal balance, and the risk of various conditions. BMI (body mass index) is the most commonly used method to define obesity, as it provides a straightforward numerical measure based on height and weight. However, it is important to note that BMI does not directly measure body fat and may not accurately reflect an individual’s health status [4,6,7].

Addressing obesity during pregnancy is crucial for several reasons and presents challenges for specialists in obstetrics and gynecology. Firstly, it has a direct impact on maternal health, increasing the risk of pregnancy complications such as gestational diabetes, pre-eclampsia, and the need for emergency cesarean delivery. Meta-analyses have shown that women classified as obese are three to four times more likely to develop gestational diabetes compared to normal-weight women [8]. The mechanisms underlying the increased risk of gestational diabetes among obese women are multifactorial, including increased insulin resistance, decreased insulin response, altered insulin transport and signaling mechanisms, and systemic inflammation, with elevated levels of inflammatory markers both before and during pregnancy [8]. Secondly, it affects fetal health by increasing the likelihood of premature birth, congenital anomalies, and childhood obesity. Not only does obesity pose clinically significant health risks to patients during and after pregnancy, but it also has negative effects with long-term health implications that require recognition and treatment [7,9]. Maternal obesity can also have adverse effects with lifelong consequences for offspring [10].

Recognizing the complexity, prevalence, and importance of addressing obesity in pregnancy is a current and insufficiently understood issue. The aim of the present study is to investigate the correlation between obesity and its implications for childbirth.

## 2. Materials and Methods

We conducted a retrospective study including 1513 patients who were referred to the 2nd Clinic of Obstetrics-Gynaecology “Dominic Stanca” Cluj-Napoca between April 2021 and January 2022 for birth assistance. Patient data were retrieved from the observation charts in the hospital archives, following informed consent. Patients with incomplete data were excluded from the study, leaving 1152 patients after database correction.

Patients were grouped into normal-weight, overweight, and obese categories using a corrected BMI value. This value was calculated by subtracting the weight of the newborn (or newborns, in the case of twin pregnancies), fetal appendages, and amniotic fluid from the patient’s weight at admission. The weight of the fetal appendages was calculated as 1/6 of the fetal weight, and the weight of the amniotic fluid was set at 800 g, based on the existing literature.

We conducted comparative analyses to explore the association between BMI and various outcomes: the method of delivery, the Apgar score at birth, the incidence of fetal distress (characterized by the presence of a non-reassuring fetal cardiac rhythm or the presence of late decelerations on cardiotocograph monitoring, a modified aspect of the amniotic fluid, or modified Doppler examination with an inverted cerebro-placental ratio), fetal birth weight, the presence of pregnancy-associated pathologies, and the occurrence of postpartum hemorrhage. Descriptive statistical analysis was performed to characterize clinical features of the patients and newborns.

The normal distribution of data was assessed for the BMI of mothers and the weight of neonates. Mean values (±standard deviation) were calculated for continuous variables, and percentage values were provided for categorical variables. The null hypothesis was assessed using the chi-square test. Patients were compared based on mean values of continuous variables using the *t*-test assuming equal variances. For categorical variables, percentages were compared using the chi-square test. Logistic regression was performed to obtain the predictive equation for fetal distress according to maternal BMI. Statistical significance was set at a *p*-value less than 0.05. Statistical analyses were performed using SPSS Statistics version 27.0 (SPSS Inc., Chicago, IL, USA, 2020).

## 3. Results

The body mass index (BMI) was calculated by correcting the parturient’s weight measured at admission (Wpa) according to the following formula:

Wr = Wpa − Wf − Wfa

where the following definitions apply:

1. Wr: the actual weight of the parturient;
2. Wpa: the parturient’s admission weight;
3. Wf: the weight of the newborn or newborns in the case of twin pregnancies;
4. Wfa: the weight of fetal appendages and amniotic fluid (the weight of fetal appendages was calculated as 1/6 of the weight of the newborn and the weight of amniotic fluid was set at 800 g on the basis of the literature).

**Figure 1:**
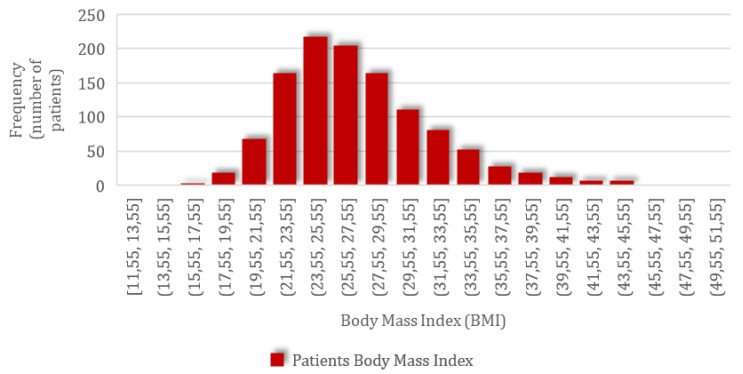
The frequency distribution of patients’ body mass index (BMI).

Following this correction, the actual parturient weight was obtained and used in the BMI calculation. For the group of 1152 patients included in the study, the mean BMI value of 27.22 kg/m^2^ (SD = 4.91; CI 95%: 26.94–27.51) was obtained, ranging from the minimum of 11.47 kg/m^2^ to the maximum value of 50.02 kg/m^2^, with a normal distribution of values according to the graph below with skewness of 0.92 and kurtosis of 1.47 (Figure 1).

**Figure 2:**
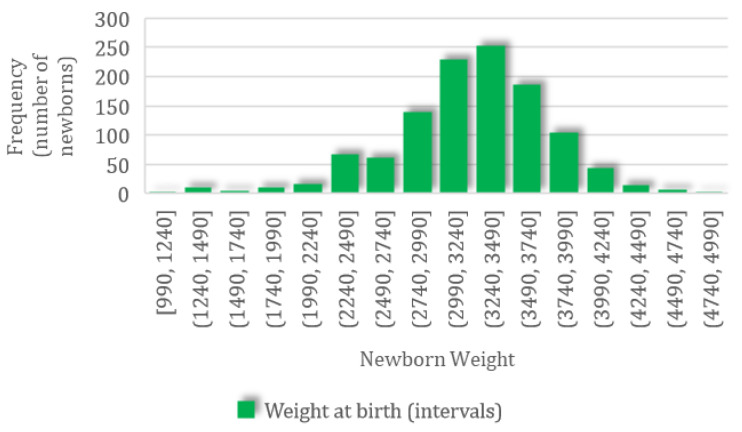
The frequency distribution of newborn body weight.

A total of 1497 newborns were recorded; for this group, the mean weight was 3210.09 (SD 545.61, CI 95%: 3182.43–3237.75) g, ranging from a minimum value of 990 g to a maximum value of 4930 g, with a normal distribution characterized by skewness of −0.53 and kurtosis of 0.97 (Figure 2).

**Figure 3:**
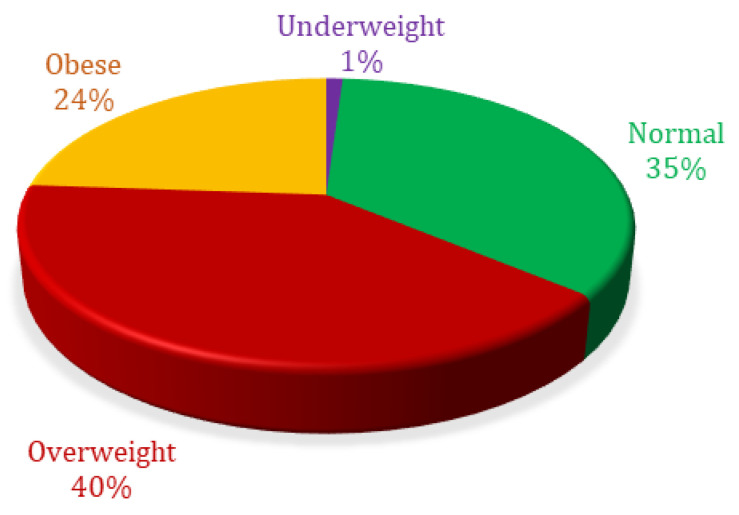
Body mass index percent distribution of patients included in analysis.

Percentage analysis based on body mass index showed 1.04% were ‘underweight’, 35.07% were ‘normal weight’, 40.02% were ‘overweight’, and 23.87% were ‘obese’ (Figure 3).

The relationship between the occurrence of fetal distress during labor and delivery and BMI was investigated, given that overweight and obese patients are known to present complications during this period. The chi-square test was employed to examine the null hypothesis regarding the influence of the pregnant woman’s body mass index on the occurrence of fetal distress at birth. Thus, by comparing normoweight pregnant women with those who are obese, as well as comparing overweight pregnant women with those who are obese, the null hypothesis was rejected (*p* < 0.05).

Of the 404 normoweight patients included in the study, 65 experienced fetal distress, while among the 275 obese patients, fetal distress during labor and delivery was identified in 62 cases, showing statistical significance (*p* < 0.05). In the comparative analysis between the group of overweight and obese patients, a total of 736 patients were included in these two categories. Among the 275 obese patients, 62 experienced fetal distress, whereas among the 461 overweight patients, 76 experienced fetal distress. Analysis of the data using the chi-square test showed statistical significance between these two groups (*p* < 0.05). These results reject the null hypothesis in both comparisons, indicating that there is a difference between normoweight vs. obese and overweight vs. obese women in the occurrence of fetal distress.

**Figure 4:**
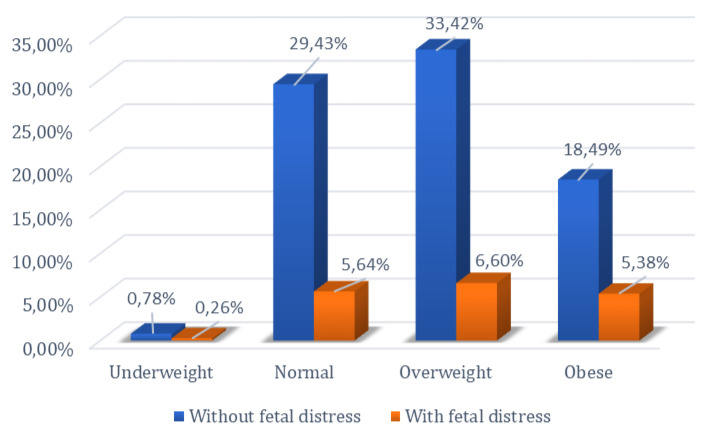
Percentage distribution of women based on BMI and presence or absence of fetal distress.

However, the null hypothesis could not be rejected when comparing normoweight with overweight pregnant women (*p* = 0.87). Among the 404 normoweight patients, 65 experienced fetal distress, and among the 461 overweight patients, 76 experienced fetal distress. The statistical analysis showed significance (*p* > 0.05), indicating that the null hypothesis for the occurrence of fetal distress could not be rejected for normoweight vs. overweight patients (Figure 4).

Considering the occurrence of fetal distress as a variable of interest, patients were divided into two groups, namely the group of patients whose newborns did not have fetal distress and the group of patients whose newborns were diagnosed with fetal distress. The statistical analysis showed a statistically significant difference in BMI between the two groups, with the “with fetal distress” group having a higher mean BMI compared to the “without fetal distress” group (Table 1).

Regarding the Apgar score at birth, the patients included in the study were also divided into two groups, namely the group of patients whose newborns had APGAR scores of 10 at 1 min postpartum and the group of patients whose newborns had APGAR scores ≤ 9 at 1 min postpartum. The statistical analysis showed a statistically significant BMI difference between the two groups, with the “score ≤ 9” group having a higher BMI compared to the “score 10” group (Table 2).

Another variable analyzed in this study was the modality of birth. The patients were divided into two groups: the group of those who gave birth vaginally and the group of patients who gave birth through cesarean section. The statistical analysis showed a statistically significant difference in BMI between the two groups, with the ‘cesarean delivery’ group having a statistically significantly higher BMI compared to the ‘vaginal delivery’ group (Table 3).

In the group of patients who went to the “Dominic Stanca” Clinic for birth assistance, we also looked for the presence of differences taking into account BMI in terms of fetal birth weight and the occurrence of postpartum hemorrhage, but using these two variables, no statistically significant differences were found.

The statistical analysis of logistic regression, using the existence or absence of fetal distress as the dependent variable, led to the following regression equation:

y = 1.3552 + 0.0062 BMI − 0.0013 Age − 0.0225 Gestational Age − 0.0001 New born weight

For the independent variables BMI, Gestational Age, and New-born Weight values of *p* < 0.001, were obtained, while for the variable Age, the result was *p* = 0.48. Thus, the null hypothesis could be excluded for the variables BMI, Gestational Age, and New-born Weight, as these independent variables could explain the risk of fetal distress at birth. The accuracy of the logistic regression function obtained was 83.03%, the precision was 65.22%, and the sensitivity was 20.45%.

## 4. Discussion

One of the topics that has gained increasing interest in recent decades is the rising incidence of obesity, now seen as an epidemic disease, mainly among young patients of reproductive age. An increase in the incidence of this pathology has been reported among women aged 20–39 years of about 33% (from 29.8% in the early 2000s to 39.7% in 2018) [8]. As for pregnant patients, data show that only two out of five women enter pregnancy with a normal body mass index, with the others being overweight or obese [8,11].

The effects of maternal obesity on pregnancy and the developing fetus have been widely studied, and systematic reviews have identified an increased incidence of pregnancy-related pathologies and complications, which include gestational diabetes [12,13]; pre-eclampsia [12,14]; gestational hypertension; depression [12,15]; cesarean section delivery; preterm birth; surgical wound infections; postpartum hemorrhage; and neonatal complications including perinatal death [12,16], macrosomia [12,17], and fetal complications (fetal abnormalities or distress and the need for care in a neonatal intensive care unit).

The results of the present study show an increase in the incidence of cesarean births with increasing BMI. This finding is consistent with other studies and data in the literature which showed that an increased maternal BMI was also associated with an increase in the number of emergency cesarean section deliveries, which was 1.30 and, respectively, 1.83 times higher in overweight and obese patients compared to normal-weight patients [18,19]. The hypothesized pathophysiological mechanism behind these higher cesarean delivery rates is that hormonally active adipose tissue may predispose patients to a reduced response to labor induction due to altered metabolic status among these types of patients with increased BMI [18,20,21]. A large study including 11,752 women found that patients classified as overweight or obese were significantly less likely to go into spontaneous labor every week of gestation after 37 weeks [22,23]. The odds of spontaneous labor decrease as BMI increases [22,24]. Obesity is associated with pregnancy progression beyond 40 weeks (aOR, 1.63; 95% CI, 1.39–1.92), 41 weeks (aOR, 1.81; 95% CI, 1.50–2.18), and 42 weeks (aOR, 1.69; 95% CI, 1.23–2.31) [22,25]. Apart from these changes, other causes of the increased rate of cesarean delivery could be the more frequent alteration in maternal status among patients with high BMI [18,26] and also the occurrence of large-for-gestational-age and macrosomic fetuses among these patients that could pose a risk for vaginal delivery due to the increased incidence of shoulder dystocia and obstetric trauma, as well as the occurrence of birth dystocia through feto-pelvic disproportion [8,18].

Another variable investigated by this study was the occurrence of fetal distress during labor and delivery. The results of the descriptive statistical analyses showed a higher frequency of fetal distress among obese patients compared to normal-weight and overweight patients. However, this difference was not identified when comparing overweight and normal-weight patients. The comparative analysis also demonstrated a higher frequency of fetal distress among patients with a high body mass index (BMI). These results are consistent with the existing literature, which indicates that maternal obesity is associated with reduced proliferation and increased apoptosis of placental villi, potentially resulting in increased susceptibility to pregnancy complications [7,27].

Animal models suggest that a high-fat diet leads to altered placental vasculature, resulting in increased placental hypoxia and oxidative stress. In these models, such changes resulted in an increase in stillbirths and a decrease in birth weight and neonatal survival [7,28], translating into the development of fetal distress. These changes and the occurrence of fetal distress are also reflected in the Apgar score at birth. The comparative analysis considering the Apgar score as a variable showed correlations between lower Apgar scores and increased maternal BMI.

Other authors have also evaluated the impact of increased BMI or excessive weight gain during pregnancy on neonatal complications in overweight and obese women. Newborns of obese mothers have a higher rate of low Apgar scores compared to those of normal-weight mothers [7,19,29,30,31], consistent with our findings. Given the higher rate of labor-related complications, it is unsurprising that newborns of obese mothers have a higher incidence of admissions to neonatal intensive care units (NICUs) with complications such as neonatal trauma and the need for incubator use [7,32]. Maternal obesity is associated with an increased overall risk of infant death, particularly neonatal deaths related to pregnancy complications or prematurity [7,33].

A retrospective cohort study of obese and overweight women found no association between excessive weight gain during pregnancy and neonatal complications (Apgar score and the need for medical care in NICUs) [34,35]. Another effect of obesity during pregnancy highlighted by previous studies is the occurrence of low-birth-weight infants (fetal weight < 2500 g). Several studies have shown relationships between pre-pregnancy obesity and a higher risk of low birth weight, although data in the literature are inconsistent [36].

The mechanisms underlying the relationship between maternal obesity and low birth weight or growth restriction are not fully understood. Placental function, maternal nutritional status, and nutrient flow from mother to fetus, in addition to genetic factors, are considered the main determinants of fetal growth [37,38,39]. Placental abnormalities and other factors (e.g., maternal pathologies) can impair fetal growth by affecting placental functions, leading to low birth weight and fetal growth restriction. Disorders accompanying obesity, such as chronic inflammation, oxidative stress, insulin resistance, neurohormone and cytokine dysregulation, and epigenetic changes, affect placental function and placental nutrient transport [40,41].

Among obese patients, opposing regulatory roles of two processes have been identified [41]. (1) Higher levels of TNF-α, IL-6, insulin, and leptin may be associated with a higher risk of fetal hypertrophy, as these markers stimulate nutrient transporter activity in the placenta [41]. (2) Conversely, maternal obesity accompanied by increased levels of IL-1 (which inhibits insulin-induced nutrient transport) and elevated soluble FMS-like tyrosine kinase (sFLT) with decreased placental growth factor (PlGF) levels, markers involved in angiogenesis, leads to reduced fetal growth [41].

Our study did not investigate the occurrence of low birth weight, but these findings open a path for future research on the influence of adipose tissue on fetal growth and the mechanisms and molecules involved in fetal growth regulation. A limitation of our study is that it did not investigate the need for newborn care in the NICU, but it identified a significant correlation between low Apgar scores at birth and maternal overweight.

Other variables, such as fetal birth weight, were also investigated in this retrospective study. The statistical analysis did not identify a significant association between maternal body mass index (BMI) and the occurrence of high birth weight or fetal macrosomia. These results are inconsistent with the literature, where numerous studies have documented a relationship between overweight and obesity and fetal growth [7,42,43,44,45]. Studies investigating this relationship have shown that obese women have an 18–26% increased chance of delivering large-for-gestational-age infants, even with the optimal control of gestational diabetes when present [7,46]. Macrosomia is associated not only with gestational diabetes and maternal obesity but also with overweight pregnancies, even among normal-weight patients, with fetal growth in this situation being 150–200 g/week [46].

The results obtained in the current study may be due to a lack of stratification of patients by obesity class when statistical analyses were performed. Another variable investigated, for which no significant association was identified, is the occurrence of maternal complications in the postpartum period, particularly postpartum hemorrhage. Previous studies have demonstrated correlations between obesity and overweight and an increased rate of postpartum complications, such as hemorrhage due to reduced uterine contractility or surgical wound infections.

The present retrospective study, unlike others, highlights the importance of the early identification of patient categories through the accurate assessment of maternal status. A strength of this study is the large number of patients included, allowing for the generalization of results. However, a limitation is that patients were selected without randomization, and potential risk factors or associated pathologies were not studied prior to selection, which could influence the results. Another limitation is the retrospective nature of data collection, limiting us to observational studies and therefore only allowing us to identify associations rather than causality.

## 5. Conclusions

Obesity is the most common health problem among women of reproductive age and requires long-term care. It can contribute to the occurrence of many pregnancy-associated pathologies and affect both mother and child during labor and delivery. Although the absolute risk of serious maternal, fetal, and newborn complications is low among women with obesity, instituting healthy eating and exercise behaviors prior to pregnancy, ideally, or as early in pregnancy as possible can minimize excessive weight gain during pregnancy. This can also help to mitigate complications arising in pregnancy, as well as long-term complications for both patients and their offspring. Future studies are needed to pinpoint the exact mechanisms by which obesity leads to pregnancy complications and influences fetal development in order to enable targeted interventions to prevent their occurrence.

## Figures and Tables

**Table 1:** Relationship between BMI and the occurrence of fetal distress.

	Fetal Distress (*n* = 1497)		*t*-Test
	Absent	Present	*p*
**Mean BMI (SD) (*n* = 1152)**	27.10 (4.83)	27.76 (5.24)	<0.05

**Table 2:** Relationship between BMI and the Apgar score at 1 minute after birth.

	Apgar Score (*n* = 1497)		*t*-Test
	10	≤9	*p*
**Mean BMI (SD) (*n* = 1152)**	27.02 (4.60)	27.61 (5.42)	<0.05

**Table 3:** Relationship between BMI and the modality of delivery.

	Modality of Delivery (*n* = 1152)		*t*-Test
	Vaginal Delivery	Cesarean Delivery	*p*
**Mean BMI (SD) (*n* = 1152)**	26.64 (4.69)	27.78 (5.05)	<0.001

## Data Availability

The raw data supporting the conclusions of this article will be made available by the authors on request.
